# Improved Eggshell Quality in Aged Hens Through Circadian Gut Microbiota and Metabolite Changes Induced by a 28-h Ahemeral Light Cycle

**DOI:** 10.3390/ani15213086

**Published:** 2025-10-24

**Authors:** Junjie Xu, Xinxin Li, Xuelu Liu, Xinling Wu, Yihao Fan, Yichun Yao, Rongcai Zhang, Dehe Wang, Yifan Chen, Erying Hao, Yanyan Sun, Jilan Chen, Hui Chen, Lei Shi

**Affiliations:** 1 College of Animal Science and Technology, Hebei Agricultural University, Baoding 071001, China; 13485949550@163.com (J.X.); 18336262159@163.com (X.L.); 15175101367@163.com (X.L.); 15053317837@163.com (X.W.); 15631100913@163.com (Y.F.); yaoyichun0819@163.com (Y.Y.); 18630258005@163.com (R.Z.); theconcertevent@163.com (D.W.); chenyfchn@163.com (Y.C.); haoerying@hebau.edu.cn (E.H.); 13930259580@163.com (H.C.); 2 State Key Laboratory of Animal Biotech Breeding, Key Laboratory of Animal (Poultry) Genetics Breeding and Reproduction, Ministry of Agriculture and Rural Affairs, Institute of Animal Science, Chinese Academy of Agricultural Sciences, Beijing 100193, China; yanyansun2014@163.com (Y.S.); chen.jilan@163.com (J.C.)

**Keywords:** hen, ahemeral light cycle, eggshell quality, gut microbiota, calcium ion absorption

## Abstract

**Simple Summary:**

This study investigated how altering the light cycle affects eggshell quality in older laying hens. We compared a standard 24 h light cycle (16L:8D) with a 28 h light cycle (16L:12D), and found that the 28 h light cycle improved eggshell strength and thickness. Through analysis of gut bacteria and blood metabolites, the study showed that the 28 h light cycle increased microbial diversity and altered daily rhythms of both microbes and metabolites. These changes were linked to improved calcium absorption and support for uterine cell growth, both critical for strong eggshell formation. The findings suggest that modifying light schedules can be a practical strategy to enhance egg quality in aging hens.

**Abstract:**

The decline in eggshell quality of aged laying hens represents a major economic challenge in poultry production. While a 28 h ahemeral light cycle has been shown to improve eggshell quality, its underlying mechanism remains unclear. This study randomly assigned 260 74-week-old Hy-Line Brown laying hens to two light cycle groups, a normal 24 h cycle group (16L:8D) and a 28 h ahemeral cycle group (16L:12D). Each treatment comprised 130 hens divided into two replicate groups. The trial lasted 16 weeks. We systematically analyzed circadian rhythms of gut microbiota and serum metabolites using 16S rRNA sequencing and untargeted metabolomics. Compared with the 24 h cycle, the 28 h cycle significantly enhanced eggshell thickness by 0.04 mm and 0.02 mm, and eggshell strength by 4.19 N and 4.76 N at 79 and 84 wk, respectively. Mechanistically, the 28 h light cycle remodeled the circadian rhythms of gut microbiota, increasing their richness and diversity, and altered the rhythmic patterns of serum metabolites. We identified nine microbial genera and three hundred seventy metabolites that exhibited opposite rhythmic patterns under the two light cycles. These changes were primarily enriched in pathways related to amino acid, carbohydrate, lipid, and energy metabolism. Correlation analysis further revealed strong associations between key microbes and functional metabolites. *Weissella* promotes calcium deposition in eggshells through synergistic interactions with calcium chelators such as gluconic acid and threonine acid. Meanwhile, *YRC22* and *Paludibacter* synergistically support membrane formation substances, thereby promoting the proliferation of uterine epithelial cells and eggshell formation. Our findings indicate that the 28 h ahemeral light cycle improved eggshell quality in aged hens by remodeling the circadian rhythms of gut microbiota and metabolites, thereby synergistically enhancing calcium ion absorption and uterine tissue health. This provides a novel theoretical basis and practical direction for improving late-phase egg quality through light management strategies.

## 1. Introduction

Eggshell quality deterioration during the late laying phase poses a significant economic challenge to the poultry industry, with a decrease in strength of 8–25% compared with the peak laying period [1,2,3,4]. The breakage rate also increases threefold and causes economic damage amounting to 100 million USD [5,6]. The decline in eggshell quality can lead to breakage during collection, storage, and transportation [2]. Therefore, the degradation of eggshell quality in poultry has become a critical issue. It is essential to improve eggshell quality in the late laying period.

The light cycle plays a crucial role in regulating circadian rhythms in poultry and exerts a significant influence on eggshell quality and laying performance [7,8]. Research has demonstrated that modifying a normal 24 h light cycle (16L:8D) can influence feed intake and eggshell quality in laying hens [9,10]. While simply extending the dark phase (9L:15D) can improve eggshell hardness, it often concurrently reduces egg weight and feed intake [11]. Notably, earlier work identified the 28 h light cycle as particularly effective among various cycle lengths tested [12,13]. Our previous research confirmed that a 28 h ahemeral cycle (16L:12D) significantly improved eggshell thickness and strength in aged hens [14] and shifted the oviposition peak into the dark phase [8]. However, the mechanisms underlying these phenomena remain poorly understood.

The formation of an eggshell is a complex process that lasts approximately 20 h and is regulated by calcium ions (Ca^2+^) to drive mineralization [15]. Approximately 66% of Ca^2+^ required for eggshell formation comes from feed intake and intestinal absorption [16,17]. It has been shown that distinct variations exist in microbial circadian rhythms under different light cycles [18,19]. Studies in humans and mice have found that >15% of gut microbiota show circadian rhythmicity [20]. Previous studies have established that light cycles significantly influence both the composition and diurnal oscillations of gut microbiota. Under extended light exposure (16L), *Actinobacteria* demonstrated increased abundance in the intestines of broilers, while shorter photoperiods (8L) favored the proliferation of *Ascomycota* and *Cyanobacteria* [21]. Furthermore, a light cycle from 12L:12D to 23L:1D in birds was shown to reduce *Bacteroides* abundance while promoting *Ruminococcus* growth, a microbial shift associated with intestinal barrier impairment and compromised nutrient absorption [22,23,24,25]. However, the circadian dynamics of the gut microbiota in laying hens and their functional association with light cycle improvements in eggshell quality remain to be investigated.

Microbial alterations subsequently modulate the production of metabolites, particularly short-chain fatty acids (SCFAs) that play pivotal roles in calcium metabolism and eggshell quality [26,27]. Although these microbial metabolites are not directly influenced by light cycles, changes in their abundance and rhythm are governed by the gut microbiota [28,29]. Changes in the gut microbiota (such as *Prevotella*) and its metabolites SCFAs in laying hens induced by photoperiodic variation corroborate this regulatory relationship [19]. Notably, microbial metabolites, including SCFAs, have been demonstrated to regulate circadian rhythms in mammals [30,31,32]. These findings underscore the importance of studying synchronized microbial communities and metabolic rhythms.

Therefore, this study used 16S rRNA sequencing and serum untargeted metabolomics to elucidate the potential mechanisms underlying improved eggshell quality under a 28 h ahemeral light cycle, and explore mechanisms for enhancing eggshell quality.

## 2. Materials and Methods

### 2.1. Experimental Design and Birds

A one-factor experiment was carried out using two treatments with two rooms (replicates) in this study, and all the rooms (replicates) were completely independent of each other. At 74 wk, 260 Hy-Line Brown layers were chosen and randomly assigned to one of two groups. There were a total of 65 birds per replicate. Following one week of prefeeding with a 16L:8D cycle, 130 birds from one group were exposed to 16L:8D (normal, 24 h light cycle), whereas the other 130 birds were exposed to 16L:12D (ahemeral, 28 h light cycle). The lighting treatments lasted for 16 wk, from 74 to 90 wk.

**Figure 1:**
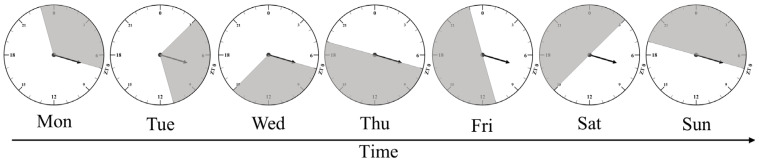
Weekly distribution map of light and dark phases in the 28 h ahemeral light cycle (16L:12D). White and black shadows indicate light and dark phases, respectively. ZT 0 means 07 am.

Light intensity was measured at bird-eye level with the photoreceptor sensor of a light meter (model: DT-1301; Shenzhen Everbest Machinery Industry Co., Ltd., Shenzhen, China). LED lamps were used with bulbs suspended 2 m above the ground, and each room was light-tight. Light intensity is controlled at 25 lux. Rooms had independent temperature and ventilation controls and were maintained at 21 °C with 50–70% relative humidity throughout this study. Temperature is monitored periodically to ensure a constant environment is maintained throughout all repeated experiments. All hens were housed in cages measuring 70 × 50 × 40 cm, located on the same level. Feed and water were provided for free and according to chicken feeding standards (Ministry of Agriculture, 2004). The basal diet composition and nutritional profiles are detailed in Table 1. The lighting schedule of the 28 h ahemeral light cycle is detailed in Figure 1.

### 2.2. Eggshell Quality and Serum Calcium

At 79 and 84 wk, 45 eggs were randomly collected from each replicate group, and their eggshell quality was assessed. We measured the weight of the egg and eggshell using a digital scale. An echo-meter and the Egg Force Reader (ESTG-1; ORKA, Herzliya, Israel) were used to measure the thickness (mm) and the strength (N) of the eggshell.

For sample collections, Zeitgeber time (ZT) denotes temporal coordinates within the light-dark cycle. ZT0 (07 am Beijing Time) was the time at which the lamps were turned on. After 4 h, this time point was designated ZT4. However, ZT28 (the next cycle’s ZT0) corresponds to 11:00 the following day. At 88 wk, twelve hens with consistent oviposition patterns were chosen from each group. At time points ZT0, ZT4, ZT8, ZT12, ZT16, ZT20, ZT24, and ZT28 (ZT4 in the normal group), the same birds took blood samples. Commercial ELISA kits from Horabio Biotechnology Co., Ltd. in Shanghai, China, and the Shenzhen Mindray BS-420 automatic biochemical analyzer were used to measure the levels of calcium in the serum.

### 2.3. Histological Observations of Uterine Tissues

At 90 wk, twelve hens from each group with consistent oviposition patterns were chosen, euthanized by cervical dislocation, and uterine tissues were collected at ZT2, ZT7, ZT12, ZT17, ZT22, and ZT27 (ZT3 in the normal group). Uterine tissue samples were fixed in 4% paraformaldehyde, embedded in paraffin wax following standard procedures, sectioned into 5 μm thick slices, and stained with hematoxylin and eosin (H&E). Sections were examined under an Olympus DP80 fluorescence microscope, with images captured at 200× magnification.

### 2.4. Cecal Contents Collection and 16S rRNA Sequences

At 90 wk, the cecal contents of the twenty-four hens mentioned above were collected at ZT2, ZT7, ZT12, ZT17, ZT22, and ZT27 (ZT3 in the normal group), and immediately frozen in liquid nitrogen for 16S rRNA sequences. For each measurement point, two hens were selected, and all hens underwent a 12 h feed withdrawal before sampling.

Genomic DNA was extracted using the OMEGA Soil DNA Kit (M5635-02, Omega Bio-Tek, Niorcross, GA, USA). The purity and concentration of the extracted DNA were detected using agarose gel electrophoresis. DNA concentration and quality were verified using a NanoDrop spectrophotometer (Thermo Fisher Scientific Inc, Shanghai, China). The V3-V4 region of the 16S rRNA gene was PCR-amplified using primers MPRK341F (5′-ACTCCTACGGGAGGCAGCAG-3′) and MPRK806R (5′-GGACTACHVGGGTWTCTAAT-3′) that included barcodes. Then, the library was sequenced on the Illumina NovaSeq platform (Illumina, San Diego, CA, USA), and 300 bp paired-end reads were generated at the Novo gene by Nanjing Personal Gene Technology Co., Ltd. (Nanjing, China). Generating an average of 103,904 ± 8079 paired-end reads per sample.

Raw data were treated and processed using the QIIME software package (version 1.7.0). Raw sequences were quality-filtered (q-score > 20), trimmed to 250 bp, and denoised using DADA2 with a 99% similarity threshold to generate amplicon sequence variants (ASVs). Removing chimeric sequences using the uchime method. Then, the representative sequences of OTU were blasted in the SILVA database (version 123). The alpha diversity and beta diversity were analyzed using the QIIME software package.

### 2.5. Serum Collection and LC-MS/MS

At 90 wk, six hens with consistent oviposition patterns were chosen from each group. At time points ZT2, ZT7, ZT12, ZT17, ZT22, and ZT27 (ZT3 in the normal group), the same birds took blood samples from the subfemoral vein. All blood samples were immediately centrifuged at 3000× *g* for 2 min at 4–8 °C to separate serum. The serum samples were stored at −80 °C.

A 20 μL of each serum sample was mixed with a 400 μL solution of methanol, acetonitrile, and water (4:4:2, *v*/*v*), vortexed, and incubated at −20 °C for 60 min for protein precipitation. Quality control (QC) samples were prepared by pooling equal aliquots from all samples. All samples were then centrifuged at 14,000× *g* and 4 °C for 20 min. A 2 μL aliquot of the supernatant was collected, vacuum-dried, and the dried residue was taken for LC-MS analysis. The supernatant was analyzed using a UPLC-Q-TOF/MS system (AB SCIEX, Shanghai, China) equipped with an ACQUITY UPLC BEH Amide column. The mobile phase consisted of (A) 25 mM ammonium acetate aqueous solution and (B) acetonitrile. Data acquisition was performed in electrospray ionization (ESI) mode with both positive and negative ion modes.

Data were pretreated using Pareto-scaling, and then multivariate statistical analysis was performed using SIMCA-P software (version 14.1, Umetrics, Umea, Sweden). Raw data were converted to mzXML format using ProteoWizard (version 3.0.8789). Peak alignment, retention time correction, and peak area extraction were performed using the XCMS program (version 3.12.0). Metabolite identification was conducted by exact mass matching (<25 ppm) and spectral matching against the HMDB, METLIN, and KEGG databases.

### 2.6. Temporal Clustering Trend Analysis

Temporal clustering analysis was performed using the Mfuzz package (version 2.58) in RStudio (version 4.3.3). The Fuzzy C-Means Clustering was applied with the following parameters: fuzzifier value of 1.25 and membership threshold set at 0.5. Determine the optimal number of clusters by observing pattern similarities in the cluster diagram. Gut microbiota and metabolites were grouped into 4 clusters and 9 clusters, respectively, based on their expression patterns. Opposite rhythmicity is defined as having clearly opposite expression trends with peak phase differences ≥12 h. And ggplot2 (version 3.4.0) was used for downstream time trend visualization, and local weighted regression (loess) was employed to smooth the trajectories of specific clusters. (*p* < 0.05).

### 2.7. Metabolite Enrichment Analysis

Based on the cluster values obtained from Temporal Clustering Trend Analysis, metabolites exhibiting opposing circadian rhythmicity were selected. Enrichment analysis was performed using the Kyoto Encyclopedia of Genes and Genomes (KEGG) database (Release 106.0), with all identified metabolites serving as the background dataset. The analysis used a hypergeometric test with the Benjamini–Hochberg false discovery rate (FDR) correction, and pathways with an adjusted *p*-value < 0.05 were regarded as statistically significant.

### 2.8. Data Analysis

All statistical analyses were performed using R software (version 4.3.3). One-way ANOVA was employed to evaluate these data. Data normality was assessed via the Shapiro–Wilk test. Multiple testing correction was performed using the FDR method. Correlation analysis between microbiota and metabolites was conducted using the Cross-Correlation Function, with data visualization generated using the ggplot2 package (R version 4.3.3). Statistical significance was defined as a *p*-value < 0.05.

## 3. Results

### 3.1. Eggshell Quality and Serum Calcium

**Figure 2:**
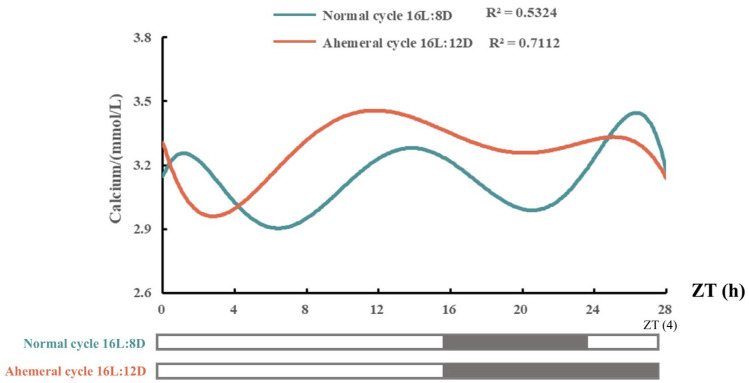
Effects of two light cycles on the serum calcium levels at 88 wk. The black shadow represents the dark period, ZT (Zeitgeber time).

As shown in Table 2, different light cycles had no significant effect (*p* > 0.05) on the laying rate, qualified egg rate, and feed-to-egg ratio (FER) in late-phase laying hens. The results for egg quality are presented in Table 3. Eggshell weight, eggshell percentage, eggshell thickness, and eggshell strength were higher (*p* < 0.05) under the 28 h ahemeral light cycle compared with the 24 h normal light cycle; the eggshell thickness and strength increased by 0.02 mm and 4.76 N, respectively. Figure 2 illustrates the effects of the two light cycles on serum calcium levels at 88 wk. While the overall diurnal pattern of serum calcium was similar under both cycles, hens exposed to the 28 h ahemeral light cycle exhibited a 4 h phase delay in the peak serum calcium concentration and achieved higher overall serum calcium levels.

### 3.2. Morphological and Histological Observations

**Figure 3:**
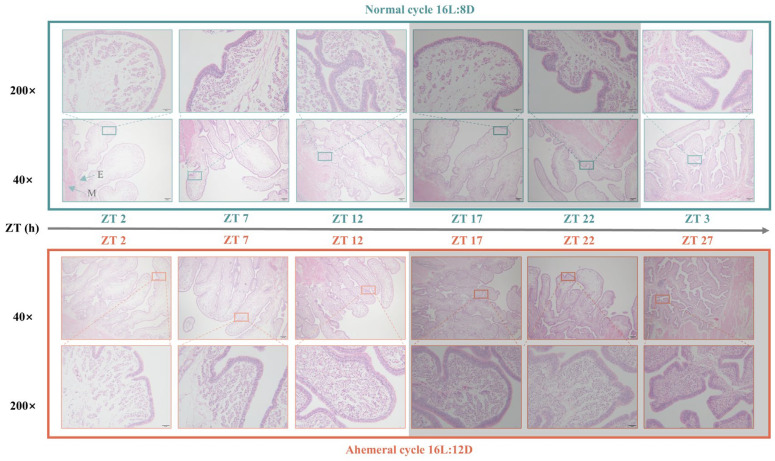
H&E stain sections of uterine tissue morphology at different time points under two light cycles in 90 wk. (40× and 200× magnification) E: endometrium; M: myometrium. The black shadow represents the dark period, ZT (Zeitgeber time).

Figure 3 presents the results of uterine H&E stain sections. Throughout the cycle, uterine folds and tubular gland cells exhibited a gradual increase under both light cycles examined. However, under the 28 h ahemeral light cycle, these structures suddenly decreased during the ZT17-22 period, followed by a sudden increase during the ZT22-27 dark phase.

### 3.3. Gut Microbiology Analysis

**Figure 4:**
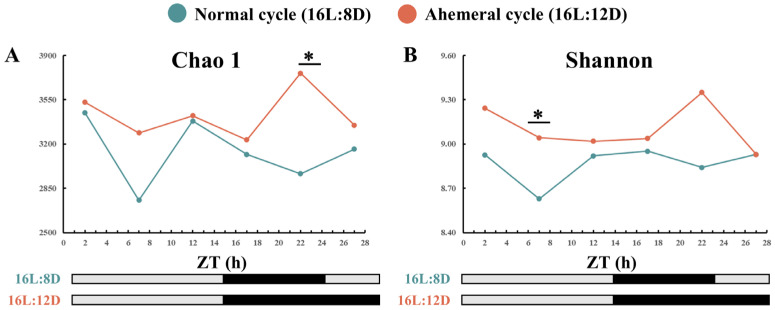
Gut microbiota α-diversity analysis, the black shadow represents the dark period, ZT (Zeitgeber time). (**A**) Chao 1 richness and (**B**) Shannon diversity. Note: ∗ *p* < 0.05.

Analysis of gut microbiota α-diversity revealed a significant increase in microbial abundance and diversity in hens under the 28 h ahemeral light cycle compared with those under the 24 h normal light cycle. Significant differences between were detected at ZT7 (Shannon, *p* < 0.05) and ZT22 (Chao1, *p* < 0.05, Figure 4).

**Figure 5:**
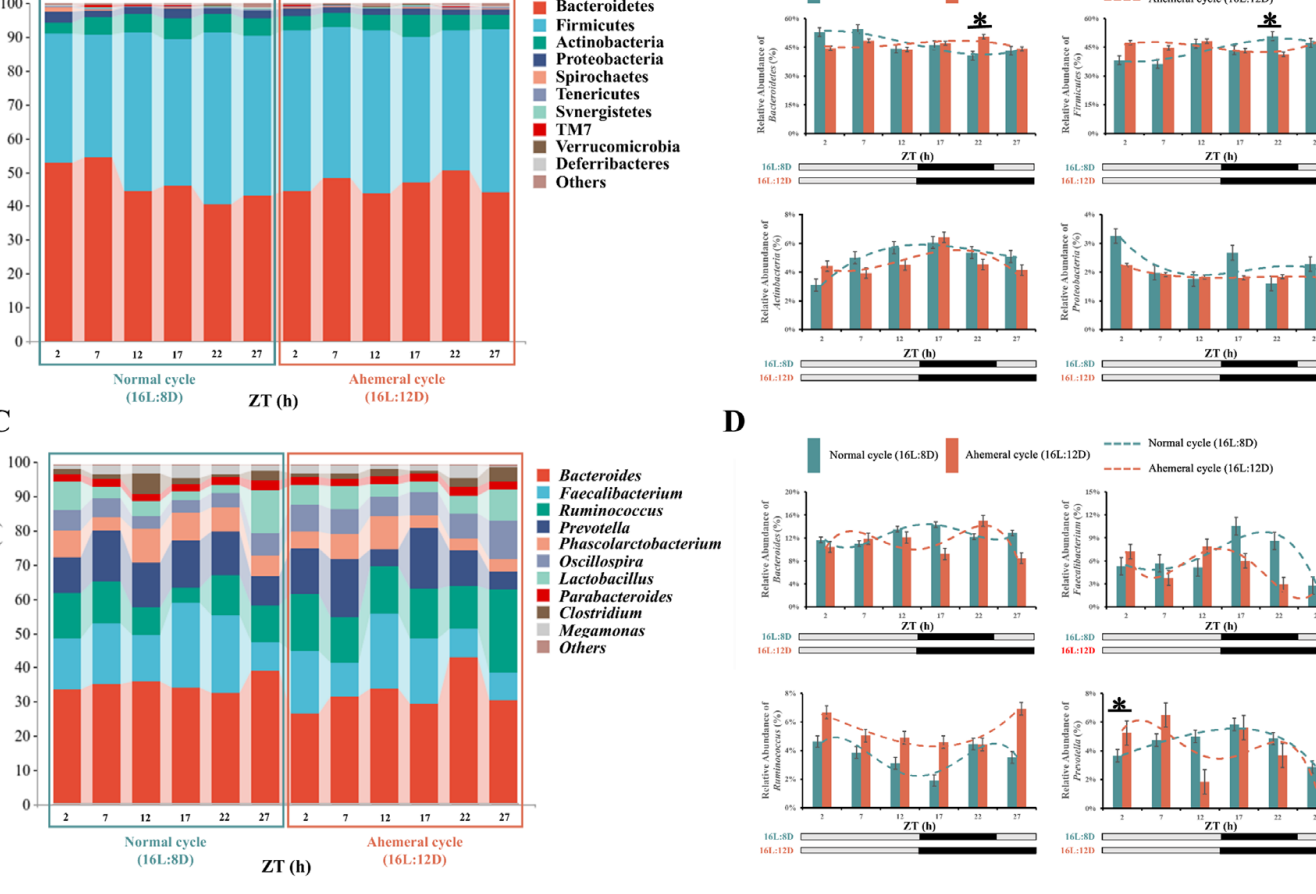
Relative abundance of gut microbiota. The black shadow represents the dark period, ZT (Zeitgeber time). (**A**) Phylum-level composition, (**B**) four dominant phyla, (**C**) genus-level composition of *Bacteroidetes* and *Firmicutes*, and (**D**) four dominant genera. Note: * *p* < 0.05.

The analysis of the community structure at the phylum level showed that both groups were predominantly composed of *Bacteroidetes*, *Firmicutes*, *Actinobacteria*, and *Proteobacteria* (Figure 5A), the relative abundances in the 24 h normal light cycle and 28 h ahemeral light cycle were 46.93:46.41%, 43.76:45.39%, 5.05:4.66%, and 2.26:1.91%, respectively, and were not different (*p* > 0.05). Notably, *Bacteroidetes* and *Firmicutes* exhibited opposing rhythmicity (Figure 5B). Under the 24 h normal light cycle, *Bacteroidetes* peaked in abundance during the light phase, while *Firmicutes* peaked during the dark phase. Conversely, under the 28 h ahemeral light cycle, *Bacteroidetes* peaked during the dark phase, whereas *Firmicutes* peaked during the light phase. Furthermore, the relative abundances of *Bacteroidetes* and *Firmicutes* at ZT22 differed significantly (*p* < 0.05).

Consequently, *Bacteroidetes* and *Firmicutes* were further analyzed at the genus level (Figure 5C). The predominant genera identified were *Bacteroides*, *Faecalibacterium*, *Ruminococcus*, and *Prevotella*. Their relative abundances in the 24 h and 28 h light cycles were 12.59:11.17%, 6.34:5.02%, 3.59:5.43%, and 4.50:4.06%. *Ruminococcus* was significantly increased by 3.20% (*p* < 0.05) under the 28 h ahemeral light cycle. *Bacteroides* displayed opposing circadian rhythmicity (Figure 5D). Under the 24 h normal light cycle, its abundance peaked during ZT12-17, but under the 28 h light cycle, peak abundance occurred during ZT2-7 and ZT22.

**Figure 6:**
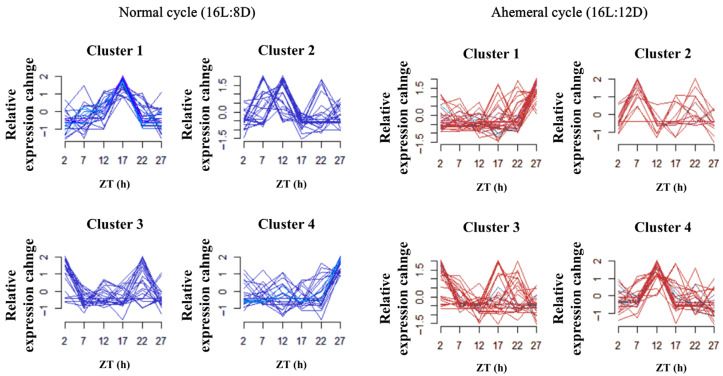
The time clustering analysis of gut microbiota under two light cycles, (**left**): normal cycle (16L:8D), (**right**): ahemeral cycle (16L:12D), gut microbiota with similar relative abundance patterns were clustered in the same cluster. Each line represents a genus of microbiota, ZT, Zeitgeber time.

**Figure 7:**
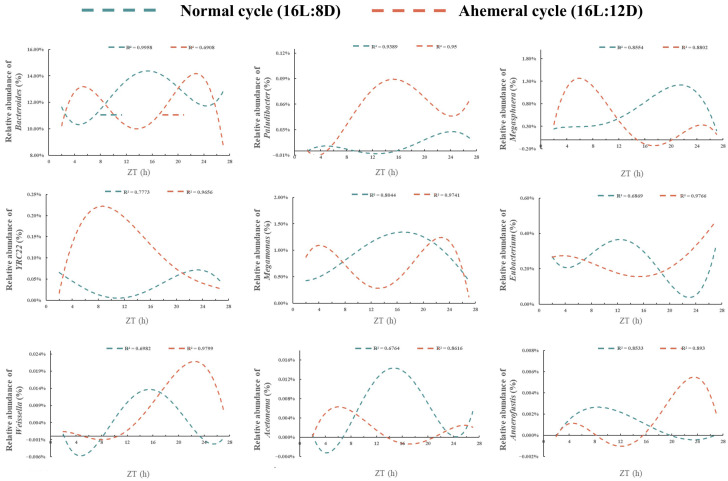
Changes in the relative abundance of gut microbiota under two light cycles. ZT, Zeitgeber time.

Through temporal clustering trend analysis, we grouped gut microbiota with similar relative abundance rhythms into the same cluster. The gut microbiota of the two groups was classified into four distinct clusters at the genus level based on *Bacteroidetes* and *Firmicutes*, respectively (Figure 6). Analysis revealed that nine gut microbiota genera exhibited opposing circadian rhythms under two light cycles (Figure 7). Among these, the relative abundance of *Weissella* showed a significant increase of 0.01% (*p* < 0.05) under the 28 h ahemeral light cycle. In contrast, the relative abundances of the other eight microbiota did not change significantly (*p* > 0.05) across the different light cycles. Five microbiota (*Megasphaera*, *Acetonema*, *YRC22*, *Paludibacter*, and *Eubacterium*) shared a similar circadian rhythmicity under the 28 h ahemeral light cycle; their peak relative abundance significantly shifted from the dark phase to the light phase. Conversely, a similar circadian rhythmicity was observed for *Weissella*, *Anaerofustis*, *Eubacterium*, and *Megamonas*, under the 28 h ahemeral light cycle; their peak relative abundance significantly shifted from the light phase to the dark phase.

These results suggest that the 28 h ahemeral light cycle may reshape the dynamic gut microbiota balance by influencing the circadian rhythm of specific genera (*Bacteroides*, *YRC22*, *Paludibacter*, etc.).

### 3.4. Metabolite Analysis

**Figure 8:**
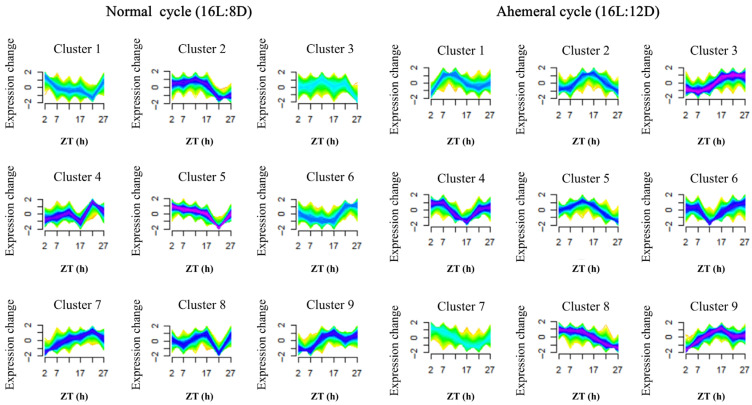
The time clustering analysis of metabolites under two light cycles, (**left**): normal cycle (16L:8D), (**right**): ahemeral cycle (16L:12D), metabolites with similar expression patterns were clustered in the same cluster. Each line represents a metabolite, ZT, Zeitgeber time.

Through temporal clustering trend analysis, we grouped metabolites with similar expression rhythms into the same cluster. Metabolites from the two groups were assigned to nine clusters, respectively (Figure 8). A total of 370 metabolites with opposing circadian rhythms were identified, of which 189 metabolites showed peak relative expression shifted from the light to dark phase at the 28 h ahemeral light cycle. The remaining 181 metabolites exhibited peak relative expression shifted from the dark to the light phase under the 28 h ahemeral light cycle.

**Figure 9:**
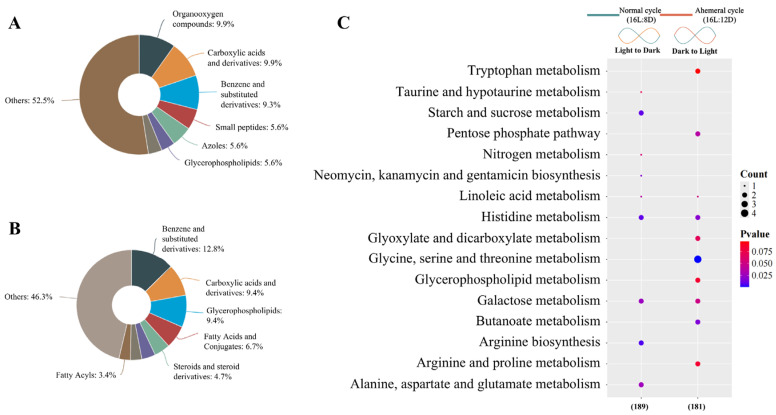
KEGG enrichment analysis of 370 circadian oscillatory rhythmicity metabolites. (**A**) 189 metabolites (higher expression from light to dark phase) constituent components, (**B**) 181 metabolites (higher expression from dark to light phase) constituent components, and (**C**) KEGG enrichment of all 370 metabolites.

The 189 metabolites with higher expression during the dark phase included organooxygen compounds, carboxylic acids and derivatives, benzene and substituted derivatives, and other related compounds (Figure 9A). The 181 metabolites with higher expression during the light phase included benzene and substituted derivatives, carboxylic acids and derivatives, glycerophospholipids, fatty acids, and conjugates, among others (Figure 9B).

Using the KEGG database as a reference, functional annotation enrichment was performed on all 370 metabolites (189/181). The 189 metabolites were mainly linked to amino acid metabolism (arginine biosynthesis, histidine metabolism, alanine, aspartate, and glutamate metabolism), carbohydrate metabolism (starch and sucrose metabolism, galactose metabolism), lipid metabolism (linoleic acid metabolism), energy metabolism (nitrogen metabolism, taurine, and hypotaurine metabolism), and other metabolic pathways (neomycin, kanamycin, and gentamicin biosynthesis). The 181 metabolites were primarily linked to amino acid metabolism (glycine, serine, threonine metabolism, histidine metabolism, arginine, proline metabolism, and tryptophan metabolism), carbohydrate metabolism (galactose metabolism, pentose phosphate pathway, glyoxylate and dicarboxylate metabolism), lipid metabolism (linoleic acid metabolism and glycerophospholipid metabolism), and energy metabolism (butanoate metabolism, Figure 9C).

### 3.5. Correlation Analysis of Gut Microbiota and Metabolites

**Figure 10:**
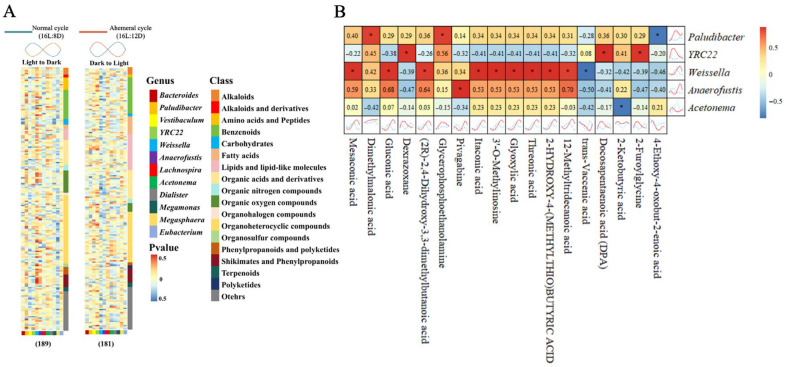
Correlation analysis between gut microbiota and metabolites. (**A**) Correlations of 189 metabolites (higher expression from light to dark phase) and 181 metabolites (higher expression from dark to light phase) with gut microbiota, (**B**) correlation heatmap between gut microbiota and metabolites. Note: * R > 0.8 or R < −0.8. Red line represents the 28 h ahemeral light cycle (16L:12D), and blue line represents the 24 h normal light cycle (16L:8D).

The metabolites were correlated individually with gut microbiota using the Cross-Correlation Function to assess associations (Figure 10A). The results indicated that *Paludibacter* was positively correlated with dimethylmalonic acid and glycerophosphoethanolamine (GPEtn, R > 0.8), but negatively correlated with 4-ethoxy-4-oxobut-2-enoic acid (R < −0.8). *YRC22* showed positive correlations with dexrazoxane, docosapentaenoic acid (DPA), and 2-furoylglycine (R > 0.8). *Weissella* displayed positive correlations with mesaconic acid, gluconic acid, (2R)-2,4-dihydroxy-3,3-dimethylbutanoic acid, itaconic acid, 3′-O-methylinosine, glyoxylic acid, threonic acid, 2-hydroxy-4-(methylthio) butyric acid, and 12-methyltridecanoic acid (R > 0.8). It also showed a negative correlation with trans-vaccenic acid (R < −0.8). *Anaerofustis* demonstrated a positive correlation with pivagabine (R > 0.8). *Acetonema* was negatively correlated with 2-ketobutyric acid (R < −0.8, Figure 10B).

## 4. Discussion

The decline in eggshell quality during the late laying phase represents a significant challenge in poultry production. While a 28 h ahemeral light cycle has been empirically shown to improve eggshell quality [8,14], the underlying physiological mechanisms, particularly those involving the gut microbiota and host metabolism, remained largely unexplored. This study integrated multi-omics and histological analyses to elucidate how a 28 h ahemeral light cycle modulates the circadian rhythms of gut microbiota and serum metabolites, thereby enhancing eggshell quality in aged laying hens.

### 4.1. Remodeling of Gut Microbial Rhythms and Diversity by the Light Cycle

This study investigated the effects of light cycles on gut microbiota circadian rhythms via 16S rRNA sequencing. The results confirmed that the 28 h ahemeral light cycle significantly altered the gut microbial composition. Notably, it enhanced both microbial abundance (Chao1 index) and diversity (Shannon index) compared with the normal 24 h light cycle, with significant differences observed at ZT7 and ZT22. This elevated microbial abundance at ZT22 may be physiologically linked to the observed shift in oviposition patterns [8]. Under the 28 h light cycle, the laying peak occurred between ZT19 and ZT21, shortly after lights-off, which likely triggered compensatory feeding and subsequently promoted microbial proliferation.

This study further characterized both the taxonomic composition and circadian rhythmicity patterns within the gut microbiota. It was found that at the phylum level, *Bacteroidetes*, *Firmicutes*, *Actinobacteria*, and *Proteobacteria* constituted the core microbiota, consistent with previous findings in laying hens [33]. Although phylum-level abundances did not differ significantly between groups, *Bacteroidetes* and *Firmicutes* exhibited opposing circadian rhythmicity. Consequently, temporal clustering analysis at the genus level was conducted for these two phyla, identifying nine key genera (*Bacteroides*, *YRC22*, *Paludibacter*, *Weissella*, *Anaerofustis*, etc.) that exhibited opposing circadian rhythmicity.

Further functional analysis of the gut microbiota revealed that *Bacteroides* are associated with feed catabolism and energy metabolism [34]. As the most abundant genus, *Bacteroides* increased in abundance from ZT2 to ZT7 and from ZT17 to ZT22. These periods corresponded to two biological processes: the eggshell formation period and the peak laying period. This suggests a potential link to energy consumption during egg laying. *YRC22* has been linked to feeding frequency [35], while *Paludibacter* demonstrated functional associations with intestinal polysaccharide catabolism pathways [36]. These genera accounted for 0.09% vs. 0.24% and 0.01% vs. 0.11% in the 24 h normal and 28 h ahemeral light cycles, respectively. Their abundance tended to increase under the 28 h ahemeral light cycle and during eggshell formation. However, our research demonstrated that hen feed consumption and FER under the two light cycles were not significantly different. These collective results may indicate more frequent feeding in laying hens under the 28 h ahemeral light cycle, supporting calcium supply and deposition, as well as energy consumption during eggshell formation.

### 4.2. Remodeling of Metabolite Rhythms by the Light Cycle

Metabolites, as substances produced or consumed during metabolic processes, play essential roles in various biological activities [37]. Serum untargeted metabolomic and temporal clustering analysis identified 370 metabolites with contrasting circadian rhythms between the two light cycles. Furthermore, KEGG enrichment analysis of metabolites with opposing trends revealed that the enriched pathways were mainly associated with amino acid, carbohydrate, lipid, and energy metabolism. The heightened activity in energy metabolism pathways is consistent with increased energy expenditure during the eggshell formation process [38]. Energy expenditure during eggshell formation primarily involves active transport mediated by Ca^2+^ pumps. These include Ca^2+^/H^+^ and Na^+^/Ca^2+^ exchangers in the uterine eggshell gland [10,39,40,41,42]. These findings suggest that higher energy use may occur because of the Ca^2+^ pump activity during eggshell formation. Meanwhile, we found a significant enrichment of butyrate and glycerophospholipid metabolism during eggshell formation. Butyrate is a key SCFA that supports epithelial cell growth and boosts energy metabolism [43,44]. Glycerophospholipid metabolism is critical for maintaining cell membrane integrity [45]. Therefore, the increased activity of these metabolic pathways may be related to the proliferation of epithelial cells and calcium pump transport.

This study also identified three Ca^2+^ chelator metabolites, gluconic acid, glyoxylic acid, and threonic acid, associated with *Weissella* (R > 0.8). These compounds chelate Ca^2+^ to form calcium gluconate, calcium oxalate, and calcium threonate [46,47,48,49]. Moreover, calcium gluconate and calcium threonate are commonly used calcium supplements to enhance intestinal Ca^2+^ absorption [50,51]. Notably, these metabolites accumulated during the dark phase and declined during the light eggshell formation period, and we observed a marked increase in blood calcium levels during the eggshell formation phase. Therefore, we hypothesized that dark-phase accumulation of gluconic acid, glyoxylic acid, and threonic acid may support the large Ca^2+^ demand during eggshell formation, leading to their significant depletion during this period. Thus, gluconic acid, glyoxylic acid, and threonic acid, as Ca^2+^ chelators, may be closely linked to Ca^2+^ absorption during eggshell formation.

### 4.3. Remodeling of Uterine Epithelial Cells by the Light Cycle

These changes observed at the metabolomic level are consistent with the structural remodeling of uterine tissue. In the present study, a gradual increase in uterine fold wall thickness and tubular gland cell numbers between ZT22 and ZT27 during eggshell formation was observed. This finding aligns with previous reports from our team [8]. Furthermore, KEGG enrichment analysis revealed a significant enrichment of the butyrate metabolism and glycerophospholipid metabolism pathways during this critical phase. These results collectively indicate active proliferation of uterine epithelial cells throughout eggshell formation.

This study also found that *YRC22* showed a strong positive correlation with DPA (R > 0.9). As a key membrane phospholipid, DPA supports cell membrane formation [52]. GPEtn, a conical lipid, regulates membrane curvature [53]. This property is linked to cell growth, division, and membrane transport functions [54]. Notably, GPEtn correlated positively with *Paludibacter* (R > 0.85). Several studies have demonstrated that gut microbiota and their metabolites (such as SCFAs) promote intestinal epithelium proliferation [55,56,57]. Furthermore, SCFAs, which are metabolites of gut microbiota, also modulate endometrial remodeling and epithelial cell integrity [58]. Collectively, these findings suggest that gut microbiota may play a role in uterine morphogenesis by supplying essential lipids for cell membrane construction and remodeling.

### 4.4. Implications and Future Perspectives

The beneficial effects observed in this study are likely attributable to the specific 28 h cycle length rather than solely to the extended dark period. This conclusion is supported by prior research identifying the 28 h cycle as optimal among various non-24 h cycles [12,13], and more importantly, by our findings of significant phase delays in circadian rhythms of serum calcium, gut microbiota, and metabolites. These phase delays indicate a reset of the host’s endogenous circadian clock. Consequently, our study advances the mechanistic understanding of photoperiod effects on eggshell quality from phenomenological observation to the level of host-microbiota interactions, providing important evidence for circadian biology research in poultry. The observed phenomenon of photoperiod reshaping microbial rhythms, which is corroborated by mammalian studies, suggests this is a conserved physiological mechanism across species [59]. These insights offer practical solutions for improving eggshell quality in aged laying hens through circadian-based lighting strategies.

## 5. Conclusions

This study demonstrates that the 28 h ahemeral light cycle improves eggshell quality in aged hens by remodeling circadian rhythms of gut microbiota and metabolism, enhancing calcium absorption and uterine function. These findings provide new insights into host-microbe circadian interactions conserved across species and offer a sustainable strategy for poultry production. This work establishes a foundation for future causal studies (e.g., fecal microbiota transplantation) and exploration of synchronized feeding strategies to optimize hen health and productivity.

## Figures and Tables

**Table 1:** Composition and nutrient levels of the basal diet.

Items	Content %
Ingredients	
Corn	62.60
Soybean meal	23.90
Wheat barn	1.80
Soybean	0.90
Limestone	8.00
CaHPO_4_	1.50
NaCl	0.30
Premix ^(1)^	1.00
Total	100.00
Nutrient levels ^(2)^	
ME/(MJ/kg)	11.24
CP	16.70
Ca	3.50
AP	0.44
Met	0.34
Lys	0.81

Notes: ^(1)^ The premix provided the following per kg of the diet: VA 10,000 IU, VD_3_ 2000 IU, VE 15 IU, VK_3_ 2.0 mg, VB_1_ 1.2 mg, VB_2_ 7 mg, VB_6_ 6 mg, VB_12_ 0.08 mg, niacin 40 mg, biotin 0.15 mg, folic acid 1.0 mg, pantothenic acid 15 mg, choline 420 mg, Cu 10 mg, Fe 60 mg, Zn 80 mg, Mn 80 mg, I 1 mg, and Se 0.3 mg. ^(2)^ CP was a measured value, while the others were calculated values.

**Table 2:** Egg production of laying hens under different light cycles from 75 wk to 90 wk.

Items	Normal Cycle 16L:8D	Ahemeral Cycle 16L:12D	SEM	*p*-Value
Laying rate/%	64.13	64.60	0.81	0.814
Qualified egg rate/%	92.90	93.58	0.23	0.145
FER	3.26	3.12	0.08	0.502

**Table 3:** Egg quality of laying hens under two light cycles was evaluated at 79 wk and 84 wk.

Week	Items	Normal Cycle 16L:8D	Ahemeral Cycle 16L:12D	SEM	*p*-Value
79 wk	Eggshell weight/g	59.41 ^a^	62.85 ^b^	0.50	0.001
	Shape index/%	1.30	1.33	0.01	0.176
	Eggshell percentage/%	8.59 ^a^	9.26 ^b^	0.09	0.001
	Yolk percentage/%	27.81	28.71	0.23	0.053
	Eggshell thickness/mm	0.31 ^a^	0.35 ^b^	0.01	0.001
	Eggshell strength/N	29.75 ^a^	33.94 ^b^	0.84	0.012
84 wk	Eggshell weight/g	58.54 ^a^	61.47 ^b^	0.39	0.001
	Shape index/%	1.33	1.33	0.01	0.877
	Eggshell percentage/%	8.87 ^a^	9.28 ^b^	0.09	0.004
	Yolk percentage/%	27.40	27.90	0.27	0.145
	Eggshell thickness/mm	0.32 ^a^	0.34 ^b^	0.01	0.001
	Eggshell strength/N	30.42 ^a^	35.18 ^b^	0.66	0.001

Note: Means within a row bearing different superscript letters ^a, b^ are significantly different (*p* < 0.05).

## Data Availability

The data presented in this study were available upon reasonable request from the corresponding author.
